# Supplementary material for: Types of anomalies in two-dimensional video-based gait analysis in uncontrolled environments
Source: PLoS Comput Biol. 2023 Jan 19;19(1):e1009989. doi: 10.1371/journal.pcbi.1009989 (PMC9851542; doi:10.1371/journal.pcbi.1009989)
Supplement: S1 Text — (DOCX) [file pcbi.1009989.s001.docx]

Types of anomalies in two-dimensional video-based gait analysis in uncontrolled environments

**Short title:** Anomalies in 2D video-based gait analysis

Yuki Sugiyama^¶1^, Kohei Uno^¶2^, Yusuke Matsui^¶*3,4^

Affiliations

^1^ Division of Physical and Occupational Therapy, Department of Integrated Health Science, Graduate School of Medicine, Nagoya University Daiko-minami, Higashi-ku, Nagoya 461-8673, Japan

^2^ Biomedical and Health Informatics Unit, Department of Integrated Health Science, Graduate School of Medicine, Nagoya University Daiko-minami, Higashi-ku, Nagoya 461-8673, Japan

^3^ Institute for Glyco-core Research (iGCORE), Nagoya University, Furo-cho, Chikusa-ku, Nagoya 464-8601, Japan

^*^ Corresponding author

Email: matsui@met.nagoya-u.ac.jp

^¶^ These authors contributed equally to this paper.

**Simulation model and accuracy evaluation**

The simulation was performed using the OU-ISIR Gait Database data. In order to generate a pseudo data set of 10,000 subjects, we selected subjects from the database who were considered to have almost no errors, and added noise according to the error types defined in Table 1. In order to select which type of error to add, the relative frequency of each of the nine error types, except for the confidence score in Table 1, was regarded as the probability of occurrence. Let $\left( X_{it}^{\star}\left[ \cdot\right],Y_{it}^{\star}\left[ \cdot\right] \right)$ be the coordinate values after adding the error.

**Undetected joint**

In OpenPose, the joint coordinates of undetected joints are output as (0,0), and the coordinate values vary near (-7,-7) due to normalization. Therefore, the joint coordinates were generated by adding a small variation based on the normal distribution to (-7,-7). The sign of the noise was determined by a Bernoulli trial (Rademacher distribution) with probability 0.5.

$$\begin{aligned} X_{it}^{\star}\left[ \cdot\right]＝-7{+ \sigma_{it}*\epsilon}_{\mathrm{it}}\left[ ・ \right]\#\left( 1 \right) \end{aligned}$$

$$\begin{aligned} Y_{it}^{\star}\left[ \cdot\right]＝-7{+ \sigma_{it}*\epsilon}_{\mathrm{it}}\left[ ・ \right]\#\left( 2 \right) \end{aligned}$$

where

$$\epsilon_{\mathrm{it}}\sim N\left( 0.1,0.1 \right) and \sigma_{it}〜Rad.$$

$\epsilon_{it}$ follows a normal distribution with mean 1.5 and variance 0.5, and $\sigma_{it}$ follows a Rademacher distribution.

**Leg length**

For the case of extremely short (or long) lower leg lengths, the true thigh length and lower leg length were generated by randomly multiplying them by a constant. First, for the right thigh length, the true right thigh length is defined by the difference between the Hip and Knee coordinates, $X_{\mathrm{it}}^{\dagger}\left[ Rk \right]-X_{\mathrm{it}}^{\dagger}\left[ Rk \right]$ and $Y_{\mathrm{it}}^{\dagger}\left[ Rk \right]-Y_{\mathrm{it}}^{\dagger}\left[ Rk \right]$. Generate the coordinates of the Knee so that the thigh length is 1.5 times or 0.5 times the Hip coordinates.

For the thigh length we have

$$\begin{aligned} X_{it}^{\star}\left[ Rk \right]＝X_{\mathrm{it}}^{\dagger}\left[ Rh \right]+\left( 1+\sigma_{it}*\epsilon_{\mathrm{it}} \right)\times\left( {X_{\mathrm{it}}^{\dagger}\left[ Rk \right]-X}_{\mathrm{it}}^{\dagger}\left[ Rh \right] \right)\#\left( 3 \right) \end{aligned}$$

$$\begin{aligned} Y_{it}^{\star}\left[ Rk \right]＝Y_{\mathrm{it}}^{\dagger}\left[ Rh \right]+\left( 1+\sigma_{it}*\epsilon_{\mathrm{it}} \right)\times\left( {Y_{\mathrm{it}}^{\dagger}\left[ Rk \right]-Y}_{\mathrm{it}}^{\dagger}\left[ Rh \right] \right)\#\left( 4 \right) \end{aligned}$$

where

$$\begin{aligned} \epsilon_{\mathrm{it}}\sim N\left( 0.5,0.1 \right) and \sigma_{it}〜Rad \end{aligned}$$

The joint coordinates of the Ankle were generated by adding the true skeletal length to $X_{it}^{\star}\left[ RK \right]$ and $Y_{it}^{\star}\left[ RK \right]$.

$$X_{it}^{\star}\left[ Ra \right]＝X_{it}^{\star}\left[ \mathrm{Rk} \right]＋({X_{\mathrm{it}}^{\dagger}\left[ Ra \right]-X}_{\mathrm{it}}^{\dagger}\left[ Rk \right])$$

$$\begin{aligned} Y_{it}^{\star}\left[ Ra \right]＝Y_{it}^{\star}\left[ \mathrm{Rk} \right]＋\left( {Y_{\mathrm{it}}^{\dagger}\left[ Ra \right]-Y}_{\mathrm{it}}^{\dagger}\left[ Rk \right] \right)\#\left( 6 \right) \end{aligned}$$

The error for the right lower leg length is calculated by$X_{\mathrm{it}}^{\dagger}\left[ Ra \right]-X_{\mathrm{it}}^{\dagger}\left[ Rk \right]$ and $Y_{\mathrm{it}}^{\dagger}\left[ Ra \right]-Y_{\mathrm{it}}^{\dagger}\left[ Rk \right]$. the frame containing the error for the right lower leg length was generated by letting Ankle coordinate values be generated as follows

$$X_{it}^{\star}\left[ Ra \right]＝X_{\mathrm{it}}^{\dagger}\left[ Rk \right]+(1+\sigma_{it}*\epsilon_{\mathrm{it}})\times({X_{\mathrm{it}}^{\dagger}\left[ Ra \right]-X}_{\mathrm{it}}^{\dagger}\left[ Rk \right])$$

$$\begin{aligned} Y_{it}^{\star}\left[ Ra \right]＝Y_{\mathrm{it}}^{\dagger}\left[ Rk \right]+\left( 1+\sigma_{it}*\epsilon_{\mathrm{it}} \right)\times\left( {Y_{\mathrm{it}}^{\dagger}\left[ Ra \right]-Y}_{\mathrm{it}}^{\dagger}\left[ Rk \right] \right)\#\left( 7 \right) \end{aligned}$$

where

$$\begin{aligned} \epsilon_{\mathrm{it}}\sim N\left( 0.5,0.1 \right) and \sigma_{it}〜Rad \end{aligned}$$

That is, $\epsilon_{\mathrm{it}}$ follows a normal distribution with mean 1.5 and variance 0.5, and $\sigma_{it}$ follows a Rademacher distribution. Similarly for the left lower limb, we can replace Rh, Rk, and Ra in the above equation with Lh, Lk, and La, respectively.

**Shoulder joint distance and COG**

For the anomalies in the shoulder joint distance and COG, we had a frame generated by adding noise to the actual frames containing the error, and inserted it into one of the consecutive frames consisting of the joint coordinates of true skeletal model.

For shoulder joint distance, we selected five frames from the real data that contained errors of typical shoulder joint distances, and randomly selected one from them. We then replaced the coordinates of all 18 joints with the true model's joint coordinates for consistency.

$$X_{it}^{\star}\left[ ・ \right]＝X_{\mathrm{it}}^{\dagger}\left[ ・ \right]{+\sigma_{it}*\epsilon}_{\mathrm{it}}\left[ ・ \right]$$

$$\begin{aligned} Y_{it}^{\star}\left[ ・ \right]＝Y_{\mathrm{it}}^{\dagger}\left[ ・ \right]{{+\sigma}_{it}*\epsilon}_{\mathrm{it}}\left[ ・ \right]\#\left( 8 \right) \end{aligned}$$

where

$$\begin{aligned} \epsilon_{\mathrm{it}}\sim N\left( 0.01,0.1 \right) and \sigma_{it}〜Rad \end{aligned}$$

We generated the data for COG in the same way.

**ROM**

For the ROM anomalous error, angular noise was added to the true value. Let's take the right hip joint as an example. First, the position coordinate of Rh is translated to (0,0). Let ($X_{it}^{\star\star}\left[ ・ \right],Y_{it}^{\star\star}\left[ ・ \right]$) be the arbitrary coordinates that have been translated. At this time

$${(X}_{it}^{\star\star}\left[ Rh \right],Y_{it}^{\star\star}\left[ Rh \right])=\left( 0,0 \right)$$

$$\begin{aligned} \left( X_{it}^{\star\star}\left[ Rk \right],Y_{it}^{\star\star}\left[ Rk \right] \right)=\left( X_{it}^{\dagger}\left[ Rk \right]-X_{it}^{\dagger}\left[ Rh \right],Y_{it}^{\dagger}\left[ Rk \right]-Y_{it}^{\dagger}\left[ Rh \right] \right)\#\left( 9 \right) \end{aligned}$$

Put the angle to be rotated as noise as follows

$$\theta\sim N\left( 30,2 \right) and \sigma_{it}〜Rad$$

Now, let ${(X}_{it}^{\star\star}\left[ Rh \right],Y_{it}^{\star\star}\left[ Rh \right])=$(0,0) and ${(X}_{it}^{\star\star}\left[ Rk \right],Y_{it}^{\star\star}\left[ Rk \right])=\left( X_{it}^{\dagger}\left[ Rk \right]-X_{it}^{\dagger}\left[ Rh \right],Y_{it}^{\dagger}\left[ Rk \right]-Y_{it}^{\dagger}\left[ Rh \right] \right)$ be rotated by $\theta$ without changing the length between Rh and Rk. Let ${(X}_{it}^{\theta}\left[ Rk \right],Y_{it}^{\theta}\left[ Rk \right])$ be the coordinates of Rk after the $\theta$-rotation. Then, to revert to the original coordinate system, we use the following procedure;

$${(X}_{it}^{\star}\left[ Rh \right],Y_{it}^{\star}\left[ Rh \right])= X_{\mathrm{it}}^{\dagger}\left[ \mathrm{Rh} \right],Y_{\mathrm{it}}^{\dagger}\left[ \mathrm{Rh} \right])$$

${(X}_{it}^{\star}\left[ Rk \right],Y_{it}^{\star}\left[ Rk \right])={(X}_{it}^{\theta}\left[ Rk \right]+X_{\mathrm{it}}^{\dagger}\left[ \mathrm{Rh} \right],Y_{it}^{\theta}\left[ Rk \right]-Y_{\mathrm{it}}^{\dagger}\left[ \mathrm{Rh} \right]$)

In this way, we can obtain the coordinates where Rk is rotated $\theta$ compared to the true coordinates. In case of the left hip joint, we just replace Rh and Rk with Lh and Lk, respectively. Also, for anomalies in the right knee joint, replace Rh and Rk with Rk and Ra, respectively, and for the left knee joint, replace Lk and La.

**Ankle joint distance**

Either under- or overestimated ankle joint distance is selected by Bernoulli trials with probability 0.5, and generated by adding random numbers with normal distribution. In actual data, the anomaly was often observed continuously in all frames of the gait cycle in individuals with an underestimated ankle joint distance, while in individuals with an overestimated ankle joint distance, the anomaly was often observed in one specific frame. In other words, when generating the case of ankle joint distance underestimation,

$$X_{it}^{\star}\left[ Rk \right]＝X_{i all}^{\dagger}\left[ Rk \right]\times\epsilon_{i all}\left[ Rk \right]$$

$$X_{it}^{\star}\left[ Ra \right]＝X_{i all}^{\dagger}\left[ Ra \right]\times\epsilon_{i all}\left[ Ra \right]$$

$$X_{it}^{\star}\left[ Lk \right]＝X_{i all}^{\dagger}\left[ Lk \right]\times\epsilon_{i all}\left[ Lk \right]$$

$$X_{it}^{\star}\left[ La \right]＝X_{i all}^{\dagger}\left[ La \right]\times\epsilon_{i all}\left[ La \right]$$

where

$$\epsilon_{\mathrm{it}}\sim N\left( 0.1,0.5 \right)$$

and if the ankle joint distance is large, then

$$X_{it}^{\star}\left[ Rk \right]＝X_{it}^{\dagger}\left[ Rk \right]\times\epsilon_{it}\left[ Rk \right]$$

$$X_{it}^{\star}\left[ Ra \right]＝X_{it}^{\dagger}\left[ Ra \right]\times\epsilon_{it}\left[ Ra \right]$$

$$X_{it}^{\star}\left[ Lk \right]＝X_{it}^{\dagger}\left[ Lk \right]\times\epsilon_{it}\left[ Lk \right]$$

$$X_{it}^{\star}\left[ La \right]＝X_{it}^{\dagger}\left[ La \right]\times\epsilon_{it}\left[ La \right]$$

where

$\epsilon_{\mathrm{it}}\sim N\left( 5,0.5 \right)$.

Here we denote all frames by “$all$”.

**Time transition**

For anomalies related to the time series transition of frames, we added noise that follows a normal distribution such that the change between frames is over or underestimated.

$$X_{it}^{\star}\left[ \cdot\right]＝X_{\mathrm{it}}^{\dagger}\left[ \cdot\right]{+\sigma}_{it}\times\epsilon_{\mathrm{it}}$$

$${Y_{it}^{\star}\left[ \cdot\right]＝Y}_{\mathrm{it}}^{\dagger}\left[ \cdot\right]{+\sigma}_{it}\times\epsilon_{\mathrm{it}}$$

where

$$\epsilon_{\mathrm{it}}\sim N\left( 1.5,0.5 \right) and \sigma_{it}〜Rad$$

**Side of legs**

Errors in which the lower limbs (hip, knee, and ankle joints) are swapped between left and right occur mainly when the left and right lower limbs overlap when viewed from the right side. Assuming that they are swapped at time $t=t^{'}$, we performed an operation to swap the $\mathrm{XY}$ coordinates in all frames at time $t^{'}\leq t$.

$X_{it}^{\star}\left[ Rh \right]＝X_{\mathrm{it}}^{\dagger}\left[ \mathrm{Lh} \right]$ *,* $Y_{it}^{\star}\left[ Rh \right]＝Y_{\mathrm{it}}^{\dagger}\left[ \mathrm{Lh} \right]$

$X_{it}^{\star}\left[ Lh \right]＝X_{\mathrm{it}}^{\dagger}\left[ \mathrm{Rh} \right]$ *,* $Y_{it}^{\star}\left[ Lh \right]＝Y_{\mathrm{it}}^{\dagger}\left[ \mathrm{Rh} \right]$

$X_{it}^{\star}\left[ Rk \right]＝X_{\mathrm{it}}^{\dagger}\left[ \mathrm{Lk} \right]$ *,* $Y_{it}^{\star}\left[ Rk \right]＝Y_{\mathrm{it}}^{\dagger}\left[ \mathrm{Lk} \right]$

$X_{it}^{\star}\left[ Lk \right]＝X_{\mathrm{it}}^{\dagger}\left[ \mathrm{Rk} \right]$ *,* $Y_{it}^{\star}\left[ Lk \right]＝Y_{\mathrm{it}}^{\dagger}\left[ \mathrm{Rk} \right]$

$X_{it}^{\star}\left[ Ra \right]＝X_{\mathrm{it}}^{\dagger}\left[ \mathrm{La} \right]$ *,* $Y_{it}^{\star}\left[ Ra \right]＝Y_{\mathrm{it}}^{\dagger}\left[ \mathrm{La} \right]$

$X_{it}^{\star}\left[ La \right]＝X_{\mathrm{it}}^{\dagger}\left[ \mathrm{Ra} \right]$ *,* $Y_{it}^{\star}\left[ La \right]＝Y_{\mathrm{it}}^{\dagger}\left[ \mathrm{Ra} \right]$

**・Grounding**

This is the case where neither ankle joint is present where the ground is expected to be present when Ne is set to (0,0). To reproduce this situation, we introduce an error that causes both ankle joints to deviate from the ground position. We considered noise such that the position of the lower limb is on average twice or 0.5 times the true skeletal length, and generated the anomaly of the lower limb according to Bernoulli trials with probability 0.5.

$$X_{it}^{\star}\left[ Ra \right]＝X_{\mathrm{it}}^{\dagger}\left[ Ra \right]\times\sigma_{it}\times\epsilon_{\mathrm{it}}$$

$$Y_{it}^{\star}\left[ Ra \right]＝Y_{\mathrm{it}}^{\dagger}\left[ Ra \right]\times\sigma_{it}\times\epsilon_{\mathrm{it}}$$

where

$$\epsilon_{\mathrm{it}}\sim N\left( 0.5,0.5 \right) if \epsilon_{\mathrm{it}}=-1$$

$$\epsilon_{\mathrm{it}}\sim N\left( 2,0.5 \right) \epsilon_{\mathrm{it}}=1$$

$$\sigma_{it}〜Rad$$
