# Supplementary figures and images for: Types of anomalies in two-dimensional video-based gait analysis in uncontrolled environments
Source: PLoS Comput Biol. 2023 Jan 19;19(1):e1009989. doi: 10.1371/journal.pcbi.1009989 (PMC9851542; doi:10.1371/journal.pcbi.1009989)

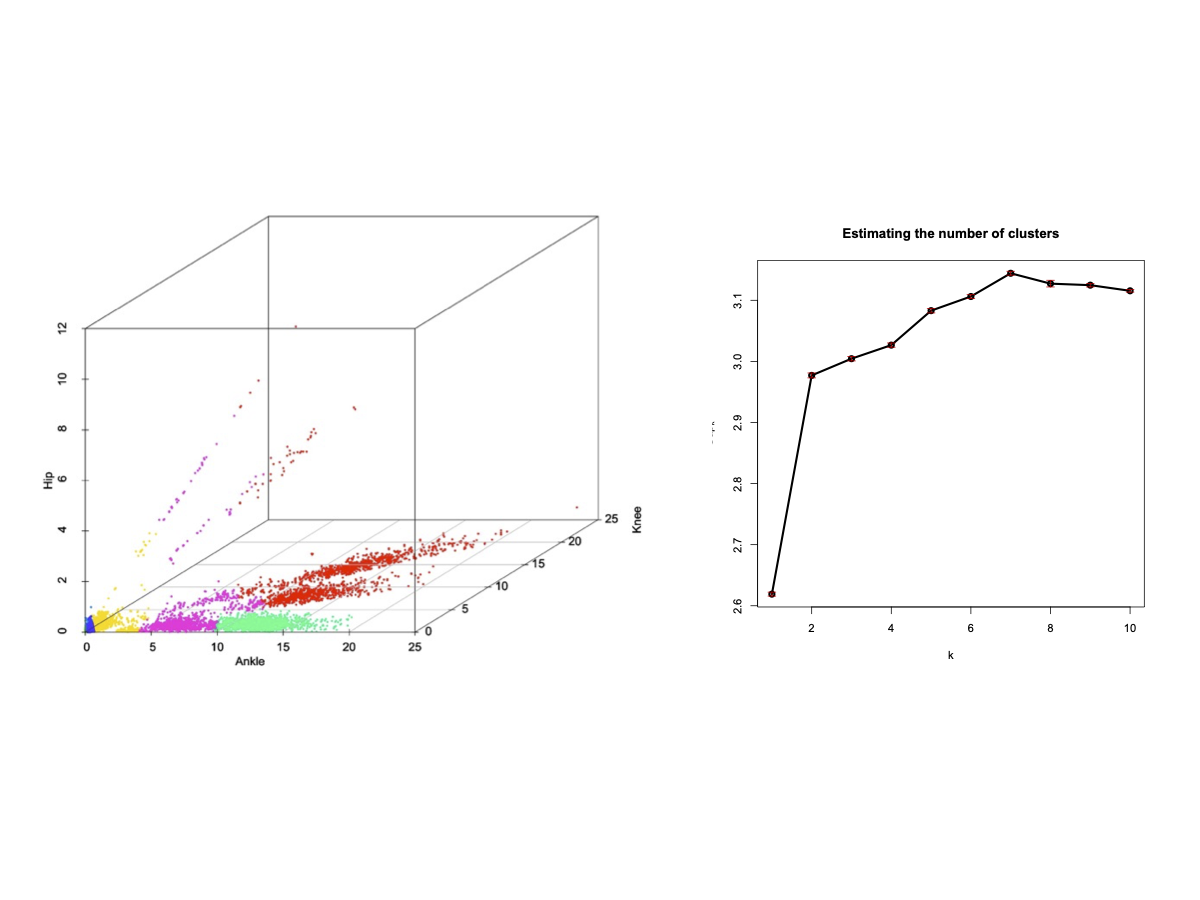

Supplement: S1 Fig — The left panel shows the coordinate values of (X,Y,Z) = (Ankle, Knee, Hip). The best cluster based on the k-means method using the gap static (right panel) is shown by color coding. Clusters very close to the origin were used as normal measurement samples. (TIFF) [file pcbi.1009989.s002.tiff]

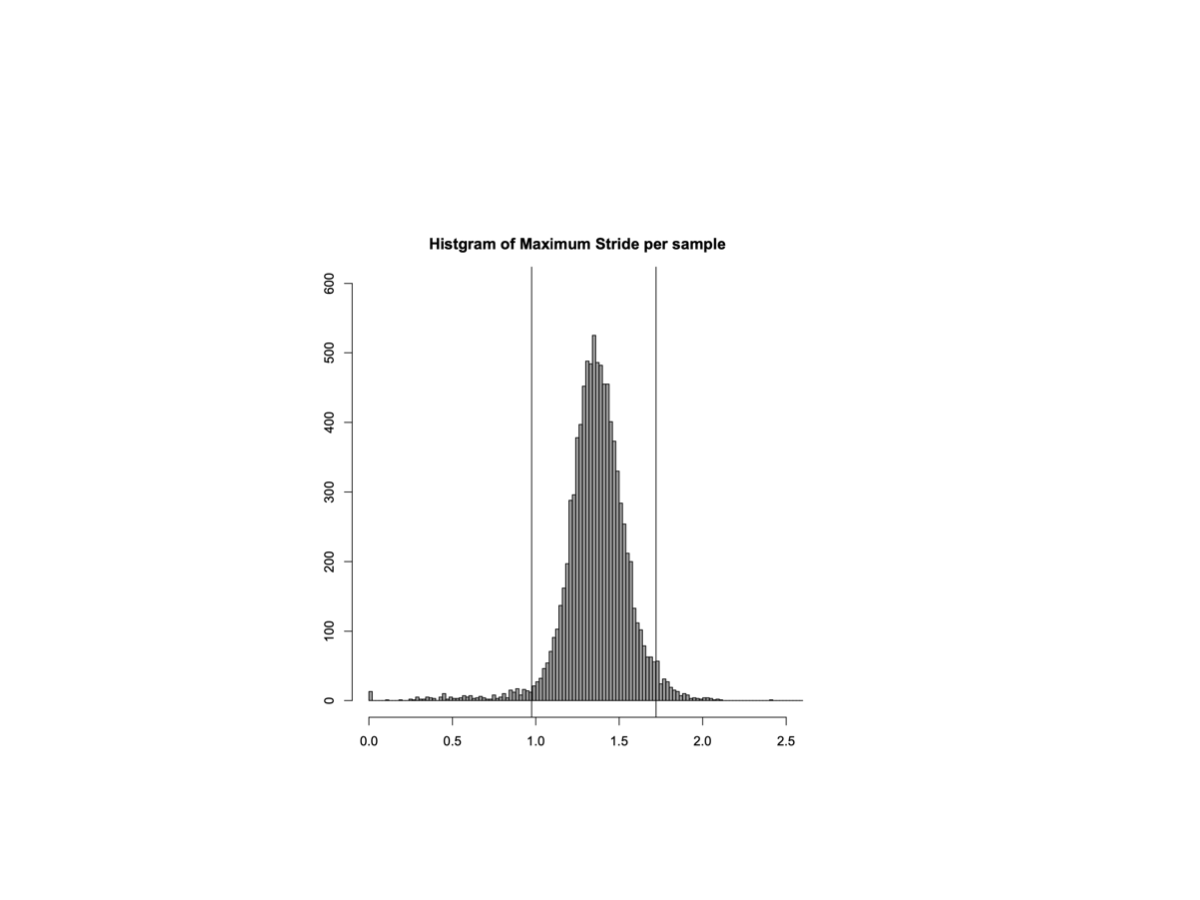

Supplement: S2 Fig — Maximum ankle joint distance within one gait cycle for each subject. To illustrate the distribution clearly, skeletal length errors due to undetected sites are excluded. (TIFF) [file pcbi.1009989.s003.tiff]

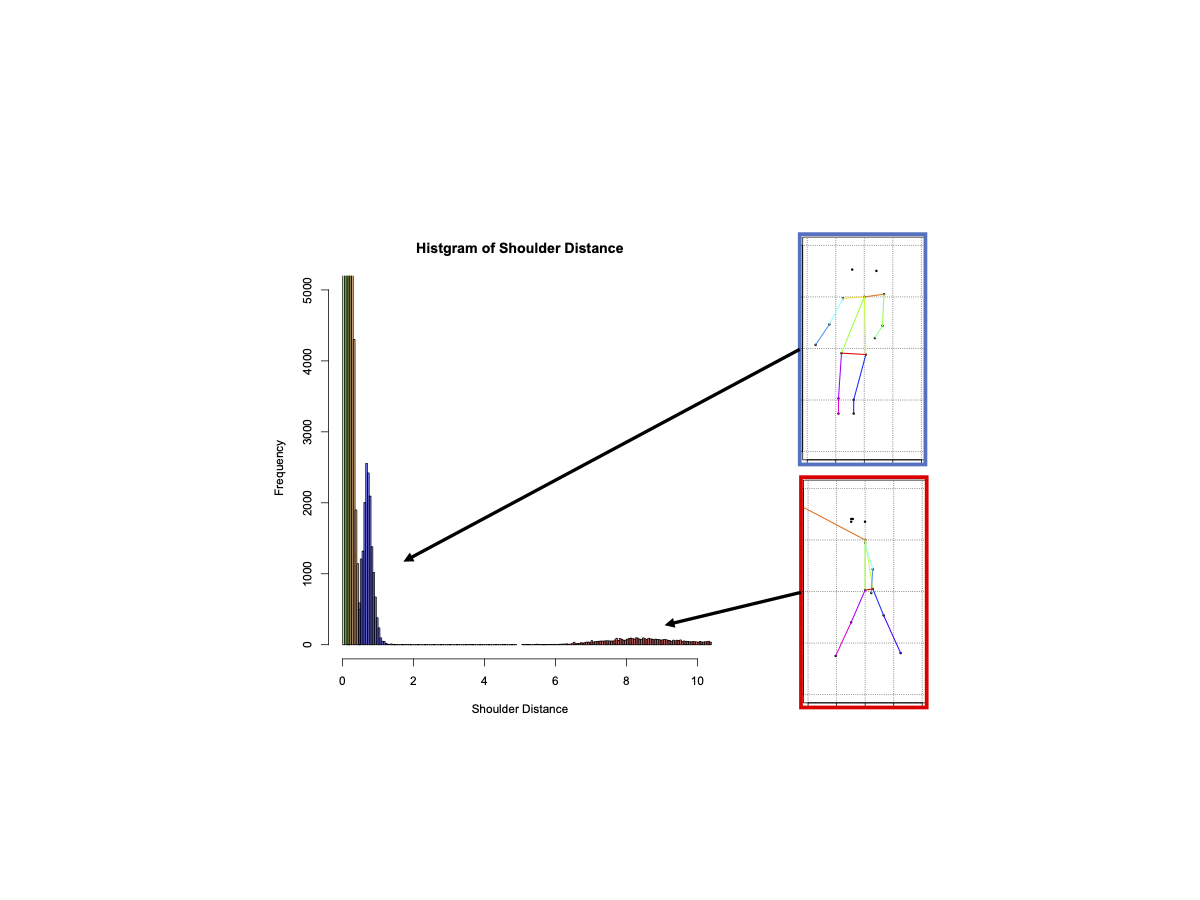

Supplement: S3 Fig — Based on clustering, the four groups were further subdivided into four group each. The group with slightly larger shoulder joint distance (blue, the third group from the left in the histogram) and the group with extremely large shoulder joint distance (red, the fourth group from the left in the histogram) were considered to have abnormal errors. (TIFF) [file pcbi.1009989.s004.tiff]

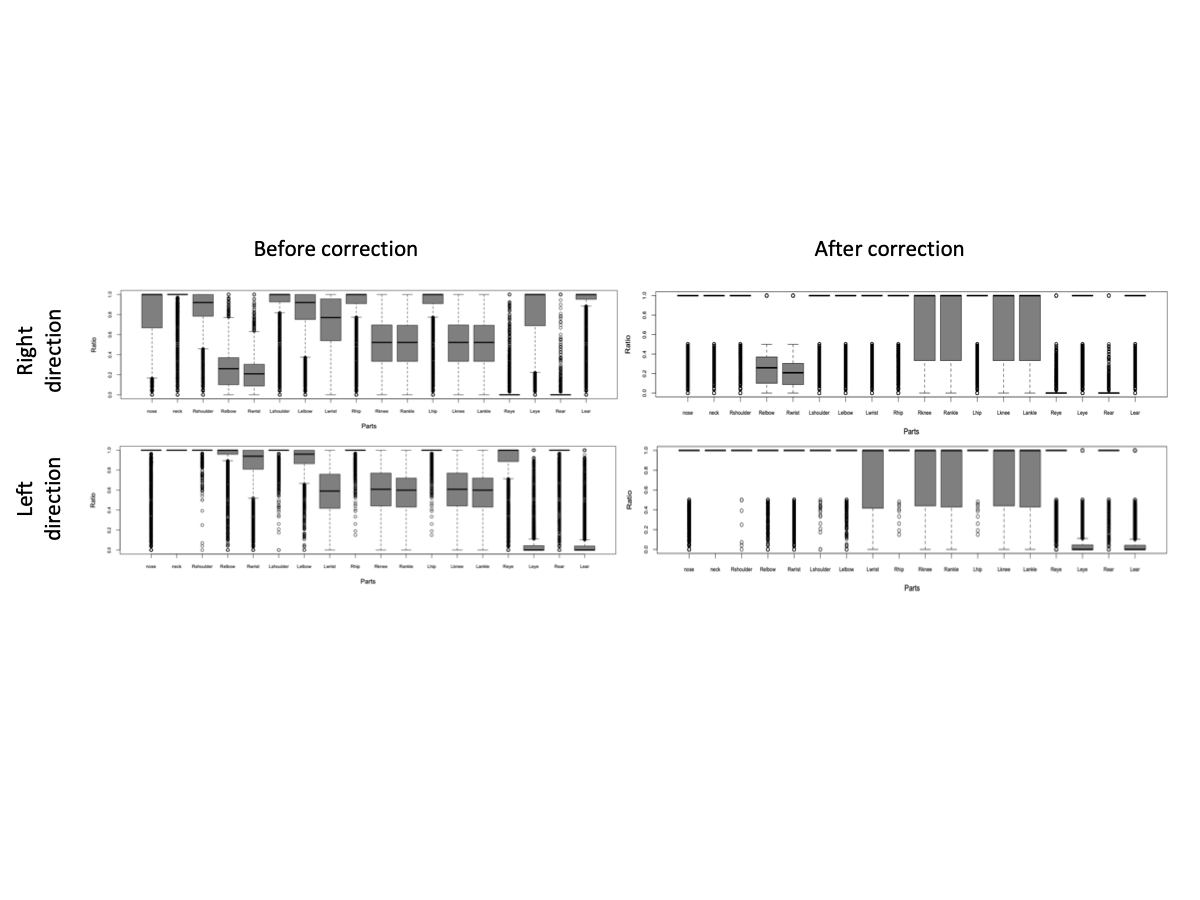

Supplement: S4 Fig — The rate of recovery before and after anomaly correction for each part, with workflow. The top and bottom rows show the accuracy for all joints during walking in the right and left directions, respectively, and the left and right rows show the accuracy before and after correction, respectively. (TIFF) [file pcbi.1009989.s005.tiff]

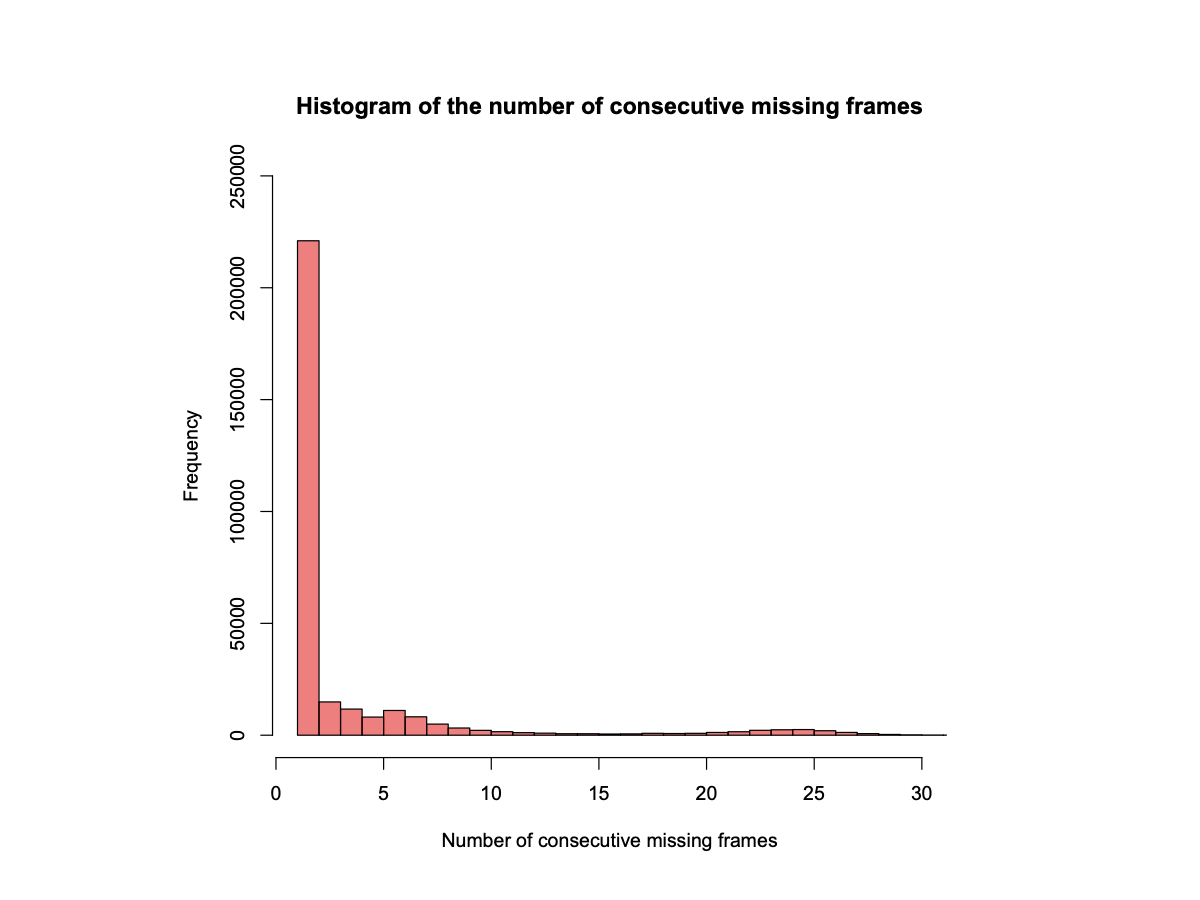

Supplement: S5 Fig — Histogram of the number of consecutive anomaly frames for all samples is shown. The samples with a number of consecutive anomalous frames over 20% of the total number of frames were excluded. (TIFF) [file pcbi.1009989.s006.tiff]
