# Supplementary material for: Types of anomalies in two-dimensional video-based gait analysis in uncontrolled environments
Source: PLoS Comput Biol. 2023 Jan 19;19(1):e1009989. doi: 10.1371/journal.pcbi.1009989 (PMC9851542; doi:10.1371/journal.pcbi.1009989)
Supplement: Attachment — Submitted filename: review_comment_add_response.docx [file pcbi.1009989.s007.docx]

Types of anomalies in two-dimensional video-based gait analysis in uncontrolled environments

Reviewer #1:

We appreciate your valuable comments. We have incorporated changes to reflect the suggestions you have provided. Please see our point-by-point responses to your comments and concerns below. In our answers, we used the line numbers. Please note that these correspond to the revised version of the manuscript, which has tracked changes denoting where the text has been changed.

INTRODUCTION

Q1-1. In the paragraph spanning lines 64-72, the authors introduce some issues with using pose estimation for gait analysis (e.g., false detection of multiple people in the image). However, previous studies (including the cited study by Stenum et al) have offered solutions for some of these issues. Given that the authors’ primary goal in this study is to focus on unconstrained environments, I suggest that they identify specifically the issues that remain with respect to unconstrained environments in this paragraph.

A1-1. Thank you for your important remarks; we have added and revised the following text in L.105-107:

“The workflow reported by Stenum et al. [16] pointed out false person detection and left-right swapping of lower limbs, but it is manual, with detection, correction, and exclusion being based on visual inspection.”

Q1-2. It also seems to me that the issues that this study aims to address are not specific to unconstrained environments. These issues may be present in unconstrained environments, of course, but they may also be present in some well-controlled environments as well. I suggest that the authors could emphasize that the analysis workflow that they present could be helpful for pose estimation-based gait analysis more broadly and not specifically in unconstrained environments.

A1-2. Thank you for your comment. We agree that this problem can arise even in well-controlled environments. Since the data in this case was verified in an uncontrolled environment, we have added the following text to the discussion.

L.526-528:

“Since our suggested category will likely include anomalous errors in the controlled environment, it is considered to be widely applicable, and not limited to pose estimation in unconstrained environments.”

Q1-3. Line 91 – I believe that the authors mean “frames per second”

A1-3. Thank you very much for pointing this out. We have corrected it as instructed.

Q1-4. There is a previous article that describes many of the issues with using currently available pose estimation algorithms for movement science (<https://arxiv.org/pdf/1907.10226.pdf>). It would be helpful if the authors could indicate in the introduction how their proposed study differs from this previous work or helps to resolve some of the issues proposed.

A1-4. Thank you for providing us with this valuable reference; Seethapathi's references are very helpful in covering many issues related to OpenPose and organizes them in a more comprehensive manner. Thus, we introduced Seethapathi et al and Stenum et al. as previous studies. We have added the following text:

L. 101-123:

“Several existing studies seem to provide a solution, although they assume a somewhat controlled data acquisition environment. Seethapathi et al. reviewed six categories of pose estimation problems that can hinder the estimation of kinematic parameters in the application of OpenPose in motion science, and suggested several possible solutions; for example, post-processing and elaboration during data acquisition, e.g., size estimation incorporating reference objects [17]. The workflow reported by Stenum et al. [16] pointed out false person detection and left-right swapping of lower limbs, but it is manual, with detection, correction, and exclusion being based on visual inspection. In an environment that can be controlled to some extent or that includes few subjects, it may be possible to devise data measurement methods or to deal with measurement errors through manual labor. However, both approaches may be limited when measuring large populations of thousands to tens of thousands of people in several different environments, such as in hospitals and other facilities.”

RESULTS

Q1-5. It is clear that the four types of anomalies identified are indeed problems for pose estimation-based gait analysis, but it is not clear how these four types of anomalies were selected. Presumably, there are other remaining issues – why/how were these four chosen in particular?

A1-5. This is a very important question that also concerns the methods and limitations of this study. The four types were not chosen a priori, but rather based on a comprehensive analysis of the database. We manually identified patterns that appeared to be anomalous errors and classified them; the four types were artificially labeled for convenience. The following text was added:

L. 160-166:

“We conducted a comprehensive analysis of the database (see Materials and Methods), while partially referring to the existing literature [16, 17], and identified four main types of anomalous errors that should be preprocessed when estimating joint coordinates during gait using OpenPose in an uncontrolled environment (Fig 1, Table 1). For convenience, the four types are categorized as anatomical, biomechanical, and physical, as well as errors due to the inherent estimation accuracy of OpenPose. Note that the categories in this study were labeled based on the patterns observed in a data-driven manner, and are used for the convenience of interpretation and to facilitate discussion.”

Q1-6. If I am understanding the results correctly, the “fix” that the authors propose for the anomalous errors is to essentially remove the data and perform gap filling, which is a rather crude solution. The authors provide some simulation work to address the reproducibility of the workflow, but this does not provide information about how accurately the gap-filled keypoints approximate ground-truth data. Comparison to some kind of ground-truth data (e.g., motion capture) would be helpful if available.

A1-6. The points you raised are very important to us as well. Since only the OpenPose public database was available for this study, we would like to acquire new data using the methods described in this study, for comparison with ground-truth data, such as Vicon, in the future. We have added the following sentence to clarify this:

L. 480-482

“Second, we didn’t compare our results to ground-truth data (e.g., motion capture) because only the OpenPose data was available in the public database. Our proposed workflow should be evaluated in further studies.”

Q1-7. Similar to my comment about the introduction, it would be helpful if the authors could contextualize their approach and findings against many of the issues proposed in the Seethapathi et al paper. It should also be mentioned as a significant limitation that we do not yet understand the accuracy of this proposed approach.

A1-7. Thank you for your suggestion.

1. We have added the following text to contextualize our work aginst the paper by Seethapathi et al.:

L. 102-105: “Seethapathi et al. reviewed six categories of pose estimation problems that can hinder the estimation of kinematic parameters in the application of OpenPose in motion science, and suggested several possible solutions; for example, post-processing and elaboration during data acquisition, e.g., size estimation incorporating reference objects [17].”

L. 484-489: “Fourth, there is a problem with OpenPose itself. As Seethapathi et al. [17] pointed out, current pose tracking algorithms do not prioritize measurement of the quantities that are important in movement science, such as three-dimensional position, velocity, and acceleration. Therefore, a new algorithm should be developed that considers important factors in kinematics, so that a more suitable system can be constructed.”

2. One significant limitation is the lack of comparison with ground-truth data, which we have mentioned in the discussion:

L. 480-482: “Second, we didn’t compare our results to ground-truth data (e.g., motion capture) because only the OpenPose data was available in the public database. Our proposed workflow should be evaluated in further studies.”

Reviewer #2: We appreciate your valuable comments. We have incorporated changes to reflect the suggestions you have provided. Please see our point-by-point responses to your comments and concerns below. In our answers, we used the line numbers. Please note that these correspond to the revised version of the manuscript, with which has tracked changes denoting where the text has been changed.

Q2-1. The paper categorizes anomaly types, but I am not sure if the proposed categorization truly identifies the actual types.

A2-1. This is a very important question that also concerns the methods and limitations of this study. We did not decide a priori which errors would be included in the analysis, but rather, we manually curated the results from an exploratory and comprehensive analysis of the database with no prior information, from various perspectives. This step was performed by a physical therapist from a professional perspective. Finally, four category labels were later assigned for ease of understanding.

This point was written in a way that was difficult to understand, so we have added a sentence at the beginning of the Result section further explaining how we identified anomaly types:

L. 164-168: “Note that the categories in this study were labeled based on the patterns observed in a data-driven manner, and are used for convenience and to facilitate discussion. Therefore, they are not based on strict anatomical, biomechanical, or physical definitions and these types are not completely independent and overlap with each other and, in some cases, are composite.”

Also, as you carefully pointed out, there are methodological limitations preventing enumeration of all the problems with OpenPose in gait analysis, for example, bias in the database itself is one. This point is described as a research limitation in the Discussion section:

L. 479-482: “However, there are several limitations. First, we did not necessarily cover all anomalies since the results are based on the analysis of a single database. Second, we didn’t compare our results to ground-truth data (e.g., motion capture) because only the OpenPose data was available in the public database. Our proposed workflow should be evaluated in further studies.”

Q2-2. The authors presents a good simulation setup. However, the robustness of their post-processing technique can be better evaluated in terms of accuracy improvement using some ground truth data coming from a motion capture system. In the current version of the text, experimental results presented in Fig.4. looks like such an evaluation, where the ground truth comparison was used. But the experiment and its purpose are not clear with lack of details. The authors should improve the text and explain this part better.

A2-2. Thank you for your important suggestion. We have added a description of the simulation, and a more detailed model has been described in Text S1.

L. 389-403.

“In order to validate the reliability of the proposed workflow, it is common to compare the results of it with the ground-truth data such as BICON. However, since there is no ground-truth data in OU-ISIR dataset; thus we conducted a simulation experiment based on actual data (Text S1) to validate the reproducibility of the workflow in this study. First, we extracted some samples from a real dataset. Second, we generated a pseudo dataset by adding various errors to these samples. The probability of occurrence of each anomalous error was calculated using relative frequencies (Table 1). We generated 10,000 subjects with 25 frames per gait cycle and evaluated the detection accuracy for each anomaly type and the reproducibility of the true joint coordinates.”

Q2-3. The authors identify 10 different anomalies. They can improve their discussion on why and how these anomalies identified with related works from the literature. Otherwise proposed categorization could be subjective. For instance, undetected-parts (Fig1) or extreme lengthening are identified under anatomical constraints. However, it is not clear why it is not categorized as one category of the estimation error. Similarly, samples identified under COG category can be also identified under anatomical constraints. Therefore, authors should better present how they define these categories and support with related studies. Lines 116-196 presents the constraint but it is not clear what is the base for identifying biomechanics or physical constraints. This should be supported with reference works. Otherwise, it is not clear why we need the proposed categorization but not the estimation error.

A2-3. Thank you for your important comment and suggestions. I agree with you about deepening the discussion, and including citation of more related research. First of all, we must say that the classification in this manuscript was introduced for convenience. Therefore, some errors belong to multiple categories. Based on our exploratory analysis and the errors reported by Stenum et. al. [16], we have enumerated several possible error types.

L. 164-168:

“Note that the categories in this study were labeled based on the patterns observed in a data-driven manner, and are used for convenience and to facilitate discussion. Therefore, they are not based on strict anatomical, biomechanical, or physical definitions and are not completely independent, as they overlap with each other and, in some cases, are composite.”

L. 207-211:

“We were unable to find any existing studies explicitly addressing and discussing this anomaly. However, in approximately 15% of the subjects, we also observed cases where the shoulder width suddenly increased even though the video was taken from the side (Fig 1c, Fig S3), and cases where the skeletal length of the lower limbs became extremely short or long before or after a certain point in time, regardless of the camera direction (Fig 1b).”

Q2-4. The Result section can be revised. The relations of subsection are getting confused. For instance, in the current version of the text, it is not explicitly written that the sections in Line 116-168 expresses different categories of constraints to detect anomalies. Otherwise, it looks like anomalies are categorize as anatomical/biomechanical etc..

A2-4.

First, the title of the first section of Results has been changed to clarify its position.

L140: “Types of anomalies” → “Overview of anomaly types”

In addition, the first paragraph of the Result section describes the structure (relationship between sections) of the subsequent sections.

L. 158-160:

“This section provides an overview of the types of anomaly errors in OpenPose measurements during gait that are presented in this paper. Individual anomaly types are discussed in detail in the section below.”

Q2-5. Some anomalies due to tracker error can be pruned based on self-evaluation of the video data, e.g. analysis on frames or consecutive frames to detect abnormal detections. Proposing such a model for post processing can be more robust than identifying various constraints with various threshold values. Therefore, the authors can add a discussion to present the advantages of their threshold-based model.

A2-5. Thank you for your important comments. We agree that it would be more flexible and robust to detect errors based on patterns such as time series information, as you have pointed out. On the other hand, since the OpenPose skeletal estimates themselves do not take into account anatomical or clinical knowledge, standard skeletal length ratios derived from existing anatomical knowledge or statistical thresholds for population-level confidence scores may be more useful.

For example, the “side of legs” anomaly (g in Fig 1.), which occurs when the left and right legs cross each other, cannot be detected by OpenPose self-evaluation, such as frame by frame analysis or measuring coordinate distances between frames. Nevertheless, this anomaly can affect joint angle estimation.

These points have been added to Discussion.

L. 495-501:

“On the other hand, this study also employed some threshold-based methods, such as standard skeletal length ratios derived from existing anatomical knowledge and confidence score thresholds based on statistical distributions. Since the OpenPose skeletal estimates themselves do not take into account anatomical or clinical knowledge, methods that combine existing knowledge may also be useful. For example, in the category of “side of legs” (g in Fig. 1), which occurs when the left and right legs cross each other, self-evaluation of OpenPose such as frame by frame analysis or coordinate distance between frames cannot detect this anomaly, although it can affect joint angle estimation.

Q2-6. The accuracies in Fig4 can be given shortly in the text with more discussion on improvements. Assuming multiple anomaly types, the authors can explain in more detail how they compute the accuracies and how they correct the values. Moreover, what is the ground truth used in this experiment?

A2-6. Thank you for your comment. Our results are based on the OpenPose database, and the ground truth for this database is unknown. Figure 4 shows the detection rate of proposed error and the performance of the correction in our proposed workflow. The "Workflow for anomaly detection and correction" section describes the way in which accuracy was calculated and anomalies corrected. We have added a sentence to explain this.

L. 317-323:

“We used a largescale database of OpenPose gait data, we examine the reliability of OpenPose estimates of joint coordinates. However, the database used in this study only contained OpenPose data, so it was not possible to evaluate the data using external criteria. Instead, using our workflow, we calculated percentages by defining joint coordinates that did not contain the anomalies shown in Table 1 as negative examples of the anomalies. The percentages of each joint determined to be normal and the percentage after correction using our workflow are summarized in Figure 4. The workflow will be described in the next section.

Q2-7. Line 374 - Line 419: In these sections, authors use some constant values. Can they give some reference study on these values? Are these values coming from some standards? Otherwise, the dataset contains various genders and age groups.

A2-7. We had made a mistake in the citations. This has now been corrected. Reference #19 provides the standard skeletal length for Asians; therefore, we defined the constant values using these standard skeletal models.

L. 482-484:

“Third, we determined threshold values (e.g., using in Eq(4)) based on a standard skeletal model [19], so gender and age groups were not considered. For more sophisticated algorithms, appropriate skeletal models should be used.”

Q2-8. Line 389: The text can be revised to clearly identify parts related to ROM, COG and ankle distance. What part is related to ROM within the text?

A2-8. Thank you for your suggestion, ROM and COG are described in the “Biomechanical constraints” subsection in the Result section. Also, as you pointed out, I have added clear indications in the method section to specify where these topics are discussed.

L. 607-608: “Here we describe abnormal detection for range of motion (ROM), which is the external angle of the axis connecting Ne and Rh (or Lh) and Rk (or Lk).”

Q2-9. Please check the abbreviations. Some includes Rh, some others include RH (similarly RK and Rk).

A2-9. Thank you for pointing this out. We have unified the abbreviations into Rh and Rk. We have also modified Eq8 in the same way.

Q2-10. Eq5: Can you please check the equation. This looks inconsistent with Eq.6

A2-10. Thank you for your comment. We have corrected the formula for consistency with Eq6.

Q.2-11. Eq7: Can you explain in the text what R_it

A 2-11. Thank you for your comment. As you pointed out, we did not include a definition for this term. Thus, we have added a definition for R_it, as follows:

L. 617–618: The joint angle degrees were set as R_it, which is defined as $\arccos\left( \cos_{it} \left[ \cdot\right] \right)\times{180^{\circ}}/\pi$

Q.2-12. Eq8: Please check the equation. Does the third component need square?

A.2-12. Thank you for your helpful remark. We have fixed this accordingly.

Q2-13. The qualities of some figures are not good to view, higher resolution would be better.

A2-13. We have uploaded higher resolution figures, but they have not appear to have good resolution in the manuscript because of the system.
